# Development and validation of a risk prediction model for dyslipidemia in community-dwelling middle-aged and older adults in China: a nationwide survey

**DOI:** 10.3389/fpubh.2024.1462483

**Published:** 2024-11-29

**Authors:** Hailong Jiang, Xiaoting Geng, Jie Shi, Chi Zhang, Chang Li, Ying Gai, Jia Mei, Shuying Li

**Affiliations:** ^1^School of Nursing, Chengde Medical University, Chengde, China; ^2^Ophthalmology, Affiliated Hospital of Chengde Medical University, Chengde, China; ^3^Department of Nursing, Chengde Nursing Vocational College, Chengde, China; ^4^Nursing Department, Tianjin First Central Hospital, Tianjin, China; ^5^Nursing Department, The First Hospital of Dalian Medical University, Dalian, China; ^6^Department of Nursing, Affiliated Hospital of Chengde Medical University, Chengde, China

**Keywords:** dyslipidemia, middle-aged and older adults, prediction model, nomogram, CHARLS

## Abstract

**Background:**

The incidence of dyslipidemia as a risk factor for many serious diseases is increasing year by year. This study aimed to construct and visualize a risk prediction model for dyslipidemia in middle-aged and older adults.

**Method:**

The subjects of our study are derived from CHARLS. Participants were allocated to training and validation groups in a 7:3 ratio at random. To identify potential predictors of dyslipidemia, we employed univariate analysis, lasso regression, and multivariate binary logistic regression analyses. A nomogram was constructed based on logistic regression results, and a ROC curve was used to evaluate its predictive performance. The accuracy and discriminatory capability were assessed using calibration curve analysis, while the net clinical benefit rate was evaluated through decision curve analysis (DCA).

**Results:**

Our study included a total of 12,589 participants, of which 1,514 were detected with dyslipidemia syndrome. Model construction: Based on the results of the logistic regression analysis of the training set, six variables were selected to construct the model, which were ranked in order of importance as comorbid hypertension, comorbid diabetes, waistline, comorbid digestive disease, place of abode, and comorbid liver disease. The ROC curve results indicated that the prediction model exhibited moderate discriminatory ability (AUC > 0.7). Additionally, the calibration curve confirmed the model's strong predictive accuracy. The decision curve analysis (DCA) illustrated a positive net benefit associated with the prediction model.

**Conclusions:**

The prediction model of dyslipidemia risk in middle-aged and older adults constructed in this study has good efficacy and helps to screen high-risk groups.

## 1 Introduction

Dyslipidemia broadly refers to abnormalities of various lipid components including elevated plasma cholesterol and/or triglyceride (TG) levels and decreased highdensity lipoprotein cholesterol (HDL-C) (1). With the rapid development of the economy causing changes in people's lifestyle and behavior, the prevalence of dyslipidemia among Chinese residents has increased significantly (2). Studies have shown that dyslipidemia is an important independent risk factor for cardiovascular diseases such as coronary heart disease and ischemic stroke (3, 4). According to the 2019 Global Burden of Disease(GBD) study (5), it was estimated that 3.78 million IHD deaths worldwide were attributed to high LDL-C, accounting for 44.3% of all IHD deaths, and 610,000 ischemic stroke deaths were attributed to high LDL-C, accounting for 22.4% of all ischemic stroke deaths. The burden of disease attributable to high LDL-C has increased rapidly since 1990, with a 46.2% increase in IHD deaths attributable to high LDL-C and a 47.8% increase in ischemic stroke deaths. It is estimated that elevated plasma cholesterol levels in the population will lead to an increase of approximately 9.2 million cardiovascular disease events in China during the period 2010–2030 seriously threatening the lives of the people (6). Therefore, it is of great significance to explore the influencing factors leading to dyslipidemia, to predict the risk of occurrence, to identify high-risk sets, to make timely interventions to reduce the occurrence of cardiovascular and cerebrovascular diseases, and improve the quality of life of residents (7).

Logistic regression modeling is a widely used method for identifying risk factors of certain diseases. Furthermore, the nomogram charts constructed on the basis of its results are more generalizable as they provide a simple graphical representation of the numerical relationship between the probability of a disease and the risk factors (8). To date, we have not identified any pertinent studies focused on developing a dyslipidemia prediction model for middle-aged and older adults in community settings. Our aim is to use Lasso-Logistic regression to analyze the China Health and Retirement Longitudinal Study 2015 data to construct and validate the prediction model for large samples and visualize it to provide a more valuable reference for the rapid identification of high-risk patients so as to formulate a more accurate treatment plan.

## 2 Materials and methods

### 2.1 Study design and data source

We used data from the China Health and Retirement Longitudinal Study (CHARLS), which was publicly available at http://CHARLS.pku.edu.cn (9). The CHARLS project seeks to gather high-quality microdata that represent households and individuals aged 45 and older in China. This data can be utilized to examine the challenges of population aging in the country and to foster interdisciplinary research on aging-related issues. The national baseline survey for CHARLS was carried out in 2011, encompassing 150 county-level units, 450 village-level units, and approximately 17,000 individuals from around 10,000 households. The cohort adhered to the Declaration of Helsinki and received approval from the Peking University Institutional Review Board (IRB0000105211015, IRB0000105213074). CHARLS 2015 data begins July 9, 2015, and ends at the end of August 2015. All participants signed the informed consent at the time of participation. Each participant has their ID to distinguish personal information. The data is of high quality and consists of a large sample, offering substantial and reliable support for the analysis presented in this article. In this cross-sectional study, we utilized data from the 2015 CHARLS survey, which encompasses demographic information, health status and functioning, biomarkers, and blood-based bioassays.

The inclusion criteria for this study were: (1) individuals aged ≥45 years; (2) possessing data on the presence or absence of dyslipidemia.

The exclusion criteria included: (1) any variables with missing values exceeding 10%.

Multiple interpolation method is used to fill the missing values (<10%). Interpolation produces 15 datasets with 20 iterations and set seed 500. This study included a total of 12,589 participants. To ensure the reliability of the model, the study cohort was randomly split into a training set (70%) and a validation set (30%) (10). Ultimately, 8,811 individuals (70%) were assigned to the training set, while 3,778 individuals (30%) were allocated to the validation set.

### 2.2 Outcome and predictor variables

Participants were classified as having or not having dyslipidemia. This study used the demographic background, health status and functioning, blood-based bioassays, and biomarkers of participants as predictor variables. Twenty-seven predictor variables were considered in this study. The demographic factors considered included age, gender, place of abode, and marital status. Gender was categorized as either male or female. Place of abode was classified as either a city or a town/village. Marital status was classified into four sets: married, divorced, widowed, and unmarried. The health status and functioning factors included smoking history, drinking history, sleep condition, activity, comorbid hypertension (hypertension), comorbid diabetes or high blood sugar (diabetes), comorbid cancer or malignant tumor (cancer), comorbid liver disease (except fatty liver, tumors, and cancer) (liver disease), comorbid kidney disease (kidney disease), comorbid stomach or other digestive disease (digestive disease), and comorbid arthritis or rheumatism (arthritis or rheumatism). The sleep condition survey was conducted by filling out a questionnaire with the title “I don't sleep well”, and when participants answered ≤ 1–2 days per week, they were considered to have good sleep status, and >1–2 days is bad. The activity survey was conducted by filling out a questionnaire titled “Do you walk >10 min per week?” to conduct the survey. The biomarker factors included waistline, BMI, and grip. The blood examination indicator included White Blood Cell Count (WBC), Mean Corpuscular Volume (MCV), Hemoglobin (HGB), C-Reactive Protein (CRP), Uric Acid (UA), Cystatin C (CysC), Hemoglobin A1c (HbA1c), Creatinine (Cr), and Blood Urea Nitrogen (BUN). According to Prediction Model Risk of Assessment Tool instructions, the transformation of continuous variables into categorical variables at the analysis stage leads to a higher risk bias in the model. Therefore, continuous variables were analyzed using the original value carryover method, while categorical variables were analyzed using the assignment method (Table 1).

**Table 1:** Categorical variable assignment.

**Categorical variable**	**Variable assignment**
Gender	Male = 1; Female = 2
Place of abode	Live in city = 1; live in village = 2
Marital status	Unmarried = 1; Widowed = 2; Divorced = 3; Married = 4
Smoking history	Yes = 1; No = 0
Drinking history	Yes = 1; No = 0
Sleep condition	Good = 1; Bad = 0
Activity	Yes = 1; No = 0
Hypertension	Yes = 1; No = 0
Diabetes	Yes = 1; No = 0
Cancer or malignant tumor	Yes = 1; No = 0
Liver disease	Yes = 1; No = 0
Kidney disease	Yes = 1; No = 0
Digestive disease	Yes = 1; No = 0
Arthritis or rheumatism	Yes = 1; No = 0

### 2.3 Statistical analysis

Statistical analyses were conducted using IBM SPSS version 25.0 and R version 4.3.3.

First, the missing data were multiplexed using the “mice” package of R, and the data were randomly divided into training and validation sets in the ratio of 7:3 using SPSS software (11). Second, Statistical descriptions and univariate analysis were performed using SPSS software. The normality test shows that all the continuous variables are skewed distribution, continuous variables were expressed as medians and quartiles, while categorical variables were reported as frequencies and percentages. Differences between sets were evaluated using the Chisquared test for categorical variables, and MannWhitney-U test for continuous variables. Univariate analysis was performed to screen for possible risk factors (12).

Third, we use the “glment” package in R software to analyze significant factors for univariate analysis by Lasso.

Last, multivariate binary logistic regression analysis using forward stepwise selection was conducted to determine the potential predictors of dyslipidemia. The statistical significance level was *P* < 0.05, and a random forest model was constructed using “RandomForest” in r software to rank the importance of the analyzed factors (13). The predictors identified from the multivariate binary logistic regression were chosen to develop a nomogram using the “rms” package in R.

**Figure 1:**
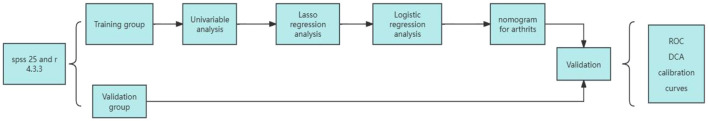
Flow diagram of analysis.

In this study, model evaluation metrics included Receiver operating characteristic (ROC), calibration curves, Decision curve analysis (DCA), Hosmer-Lemeshow tests, and accuracy. The principle of the ROC curve is to calculate the corresponding sensitivity and specificity by setting multiple critical values. The curve is then plotted with sensitivity as the vertical coordinate and 1-specificity as the horizontal coordinate to evaluate the classification performance of the model. The area under the curve (AUC) is used to quantify the model effect: an AUC value of 0.7–0.9 indicates good predictive ability, while a value above 0.9 indicates that the model has high predictive ability (14). The calibration curve was generated through 1,000 iterations of bootstrap self-sampling using the bootstrap method. The accuracy and reliability of the model in the training and validation sets can be effectively assessed by establishing calibration curves. By comparing the predicted and actual values, the calibration curves are closer to the 45-degree diagonal, indicating that the predictive ability of the model is closer to the actual results (15). Decisioncurve analysis (DCA) was also performed using the “DecisionCurve” package in R.DCA was carried out to evaluate the net clinical benefit of the model (16). The DCA curve, which introduces a loss function based on the regression prediction analysis, is used to assess the probability of the model's benefit threshold by calculating the gain and loss values of the relevant interventions and is used to illustrate the clinical utility of the model (17). The flow chart of the statistical analysis is shown in Figure 1.

## 3 Results

### 3.1 Basic information and univariable analysis of training set

A total of 12,589 participants (1,514 [12.0%] with and 11,075 [88.0%] without dyslipidemia) were selected for this study (5,834 [46.3%] males and 6,755 [53.7%] females). As indicated in Table 2, there were no differences in baseline characteristics between the training and validation sets. Univariate analysis of the variables in the training set showed that: age, place of abode, smoking history, drinking history, sleep condition, activity, WBC, MCV, HGB, CRP, UA, CysC, BUN, waistline, BMI, hypertension, diabetes, cancer, liver disease, kidneydisease, digestive diseases and arthritis or rheumatism were statistically significant (*p* < 0.05) (Table 3).

**Table 2:** Characteristics of baseline demographic and clinical indicators of the participants in the different sets.

**Variables**	**Validation set; *N* (3,778)**	**Training set; *N* (8,811)**	**χ2/t**	***P* value**
	***N*** **(%)/Media (P**_25_**,P**_75_**)**			
**Gender**			<0.001	**0.994**
Male	1,751 (46.3)	4,083 (46.3)		
Female	2,027 (53.7)	4,728 (53.7)		
**Age**	62 (54,69)	62 (54,69)	–0.615	**0.539**
**Marital status**				
Unmarried	26 (0.70)	59 (0.70)	0.985	**0.805**
Widowed	436 (11.5)	964 (10.9)		
Divorced	38 (1.0)	90 (1.0)		
Married	3,278 (86.8)	7,698 (87.4)		
**Place of abode**			0.334	**0.563**
City	628 (16.60)	1,428 (16.20)		
Town/village	3,150 (83.40)	7,383 (83.80)		
**Smoking history**			0.507	**0.447**
No	3,034 (80.30)	7,027 (79.80)		
Yes	744 (19.70)	1,784 (20.20)		
**Drinking history**			0.045	**0.832**
No	2,748 (72.70)	6,425 (72.90)		
Yes	1,030 (27.30)	2,386 (27.10)		
**Sleep condition**			0.052	**0.802**
Good	2,491 (65.90)	5,791 (65.70)		
Bad	1,287 (34.10)	3,020 (34.30)		
**Activity**			0.026	**0.873**
Yes	2,992 (79.20)	6,989 (79.30)		
No	786 (20.80)	1,822 (20.70)		
**WBC**	5.70 (4.80,6.85)	5.72 (4.74,6.90)	–0.159	**0.873**
**MCV**	91.90 (88.00,95.70)	92.00 (88.00,95.80)	–0.553	**0.580**
**HGB**	13.60 (12.57,14.80)	13.60 (12.50,14.80)	–0.569	**0.569**
**CRP**	1.39 (0.80,2.60)	1.39 (0.69,2.59)	–0.918	**0.359**
**UA**	4,80 (4.00,5.70)	4.80 (3.90,5.69)	–0.102	**0.919**
**CysC**	0.82 (0.71,0.94)	0.82 (0.71,0.95)	–1.179	**0.239**
**HbA1c**	5.80 (5.50,6.10)	5.80 (5.50,6.10)	–0.096	**0.923**
**Crea**	0.76 (0.65,0.89)	0.76 (0.66,0.89)	–0.161	**0.872**
**BUN**	14.56 (12.32,17.92)	14.84 (12.32,17.92)	–0.755	**0.450**
**Waistline**	86.00 (79.00,93.40)	86.00 (79.00,93.40)	–0.016	**0.987**
**Grip**	30.5 (24.00,38.02)	30.00 (24.10,38.00)	–0.102	**0.919**
**BMI**	23.69 (21.39,26.33)	23.71 (21.36,26.29)	–0.558	**0.577**
**Hypertension**			1.214	**0.270**
No	2,784 (73.70)	6,409 (72.70)		
Yes	994 (26.30)	2,402 (27.30)		
**Diabetes**			2.212	**0.137**
No	3,525 (93.30)	8,155 (92.60)		
Yes	253 (6.70)	656 (7.40)		
**Cancer**			0.136	**0.712**
No	3,745 (99.10)	8,728 (99.10)		
Yes	33 (0.90)	83 (0.90)		
**Liver disease**			0.021	**0.883**
No	3,650 (96.60)	8,517 (96.70)		
Yes	128 (3.40)	294 (3.30)		
**Kidney disease**			0.774	**0.379**
No	3,578 (94.70)	8,310 (94.30)		
Yes	200 (5.30)	501 (5.70)		
**Digestive disease**			0.679	**0.410**
No	3,025 (80.10)	6,998 (79.40)		
Yes	753 (19.90)	1,813 (20.60)		
**Arthritis or rheumatism**			0.366	**0.545**
No	2,667 (70.60)	6,267 (71.10)		
Yes	1,111 (29.40)	2,544 (28.90)		
**Dyslipidemia**			1.204	**0.272**
No	3,342 (88.50)	7,733 (87.80)		
Yes	436 (11.50)	1,078 (12.20)		

**Table 3:** Univariate analysis of baseline demographic and clinical indicators of the participants in the training set.

**Variables**	**Dyslipidemia (*n* = 1,078)**	**Non dyslipidemia (*n* = 7,733)**	**χ2/t**	***P* value**
	***N*** **(%)/Media (P**_25_**,P**_75_**)**			
**Gender**			1.623	**0.203**
Male	480 (44.5)	3,603 (46.6)		
Female	598 (55.5)	4,130 (53.4)		
**Age**	63 (56,69)	62 (53,69)	–4.753	**<0.001**
**Marital status**				
Unmarried	7 (0.6)	52 (0.7)	1.315	**0.725**
Widowed	107 (9.9)	857 (11.1)		
Divorced	11 (1)	79 (1)		
Married	953 (88.4)	6,745 (87.2)		
**Place of abode**			72.16	**<0.001**
City	271 (25.1)	1,157 (15.0)		
Town/village	807 (74.9)	6,576 (85.0)		
**Smoking history**			7.244	**0.007**
No	893 (82.8)	6,134 (79.3)		
Yes	186 (17,2)	1,599 (20.7)		
**Drinking history**			5.508	**0.019**
No	754 (69.9)	5,671 (73.3)		
Yes	324 (30.1)	2,062 (26.7)		
**Sleep condition**			14.458	**<0.001**
Good	653 (60.6)	5,138 (66.4)		
Bad	425 (39.4)	2,595 (33.6)		
**Activity**			2.068	**0.150**
No	873 (81.0)	6,116 (79,1)		
Yes	205 (19.0)	1,617 (20.0.9)		
**WBC**	5.98 (5.00,7.12)	5.7 (4.7,6.9)	–4.892	**<0.001**
**MCV**	91.50 (87.90,95.00)	92.00 (88.10,95.90)	–2.404	**0.016**
**HGB**	13.70 (12.70,14.90)	13.60 (12.50,14.80)	–3.109	**0.002**
**CRP**	1.89 (1.00,3.30)	1.29 (0.70,2.50)	–10.332	**<0.001**
**UA**	5.10 (4.30,6.01)	4.70 (3.90,5.70)	–7.831	**<0.001**
**CYSC**	0.86 (0.74,0.98)	0.82 (0.71,0.94)	–6.452	**<0.001**
**HbA1c**	6.00 (5.70,6.50)	5.80 (5.50,6.10)	–13.666	**<0.001**
**Crea**	0.76 (0.65,0.92)	0.76 (0.66,0.89)	–1.012	**0.312**
**BUN**	14.84 (12.32,17.92)	14.84 (12.32,17.92)	–0.100	**0.920**
**Waistline**	92.5 (85.6,99.33)	85.20 (78.30,92.40)	–19.210	**<0.001**
**Grip**	30.00 (23.78,38.9)	30.10 (24.50,38.00)	–1.103	**0.270**
**BMI**	25.73 (23.15,28.41)	23.48 (21.13,25.96)	–17.525	**<0.001**
**Hypertension**			602.199	**<0.001**
No	448 (41.6)	5,961 (77.1)		
Yes	630 (58.4)	1,772 (22.9)		
**Diabetes**			569.805	**<0.001**
No	805 (74.7)	7,350 (95.0)		
Yes	273 (25.3)	383 (5.0)		
**Cancer**			13.323	**<0.001**
No	1,057 (98.1)	7,671 (99.2)		
Yes	21 (1.9)	62 (0.8)		
**Liver disease**			42.540	**<0.001**
No	1,006 (93.3)	7,511 (97.1)		
Yes	72 (6.7)	222 (2.9)		
**Kidney disease**			50.671	**<0.001**
No	996 (89.6)	7,344 (95.0)		
Yes	112 (10.4)	389 (5.0)		
**Digestive disease**			36.559	**<0.001**
No	781 (72.4)	6,217 (80.4)		
Yes	297 (27.6)	1,516 (19.6)		
**Arthritis or rheumatism**			35.242	**<0.001**
No	684 (63.5)	5,583 (72.2)		
Yes	394 (36.5)	2,150 (27,8)		

### 3.2 Predictive model development

**Figure 2:**
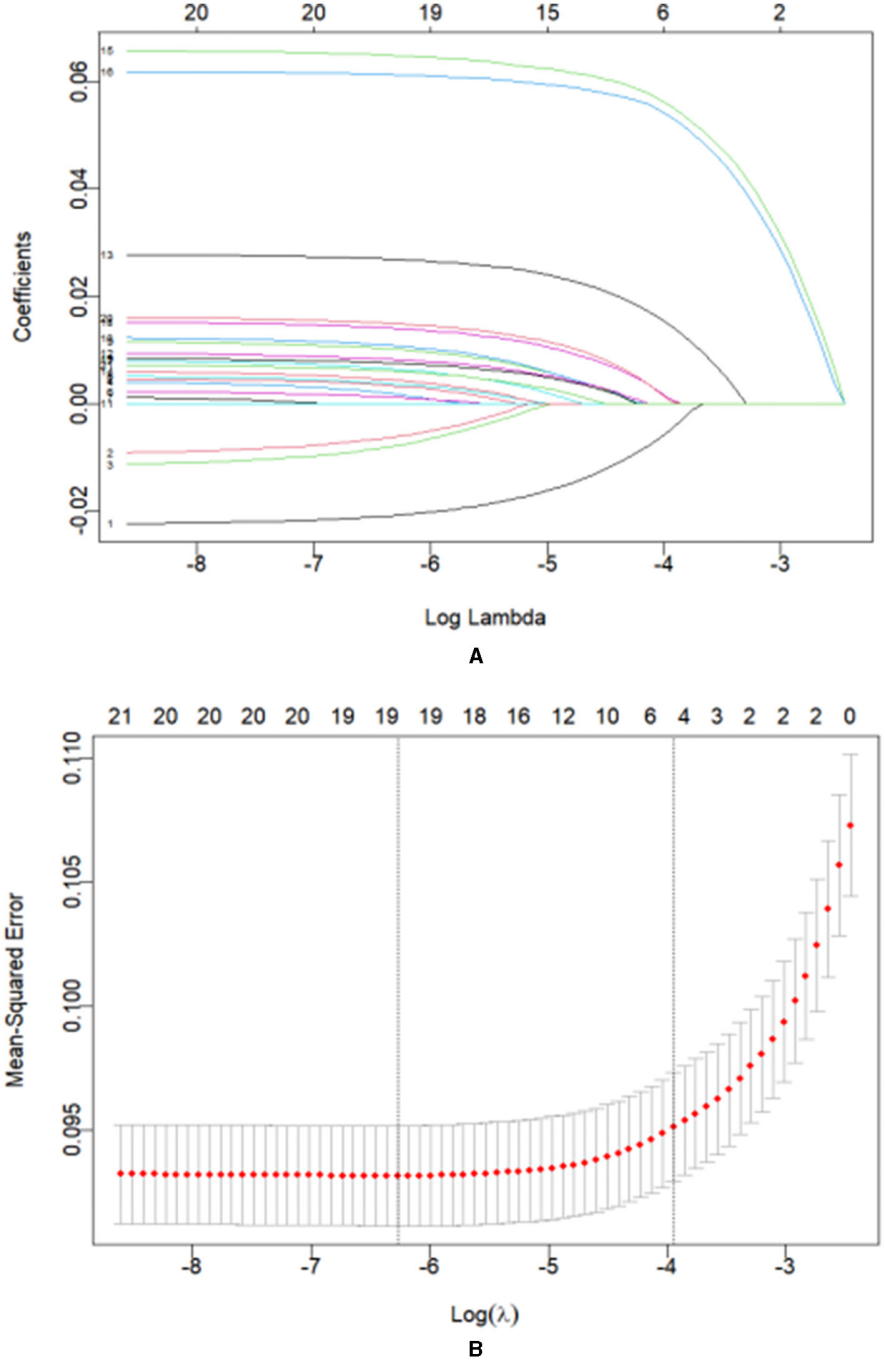
**(A)** Coefficient curves for 21 clinical features. **(B)** Lasso regression 10-fold cross-validation to select the most appropriate clinical features. A virtual vertical line at the optimal value was drawn using one SE of minimum criterion (the 1-SE criterion). Choose the minimum Lambda = 0.01934, i.e., log(Lambda) = −1.71.

Predictors were further screened using LASSO regression analysis, utilizing 10-fold cross-validation with a random seed value of 110. Non-zero coefficients were identified as potential predictors of dyslipidemia (Figures 2A, B). Select the lasso regression analysis meaningful factors including place of abode, waistline, hypertension, diabetes, liver diseases, and digestive disease. These potential factors were then incorporated into the logistic regression model. The outcomes of the logistic regression analysis are presented in Table 4.

**Table 4:** The results of the binary logistic regression analysis in the training set.

**Variables**	**B**	**OR**	**SE**	**Wald**	**95%CI**	***P* value**
Place of abode	–0.537	0.584	0.085	40.109	0.495~0.690	**<0.001**
Waistline	0.039	1.040	0.003	140.525	1.003~1.047	**<0.001**
Hypertension	1.239	3.453	0.072	295.501	2.998~3.977	**<0.001**
Diabetes	1.447	4.250	0.095	233.833	3.351~5.116	<0.001
Liver disease	0.738	2.091	0.157	22.066	1.537~2.845	**<0.001**
Digestive disease	0.517	1.677	0.082	39.564	1.428~1.970	**<0.001**
Constants	–5.295	0.005	0.341	240.843		**<0.001**

**Figure 3:**
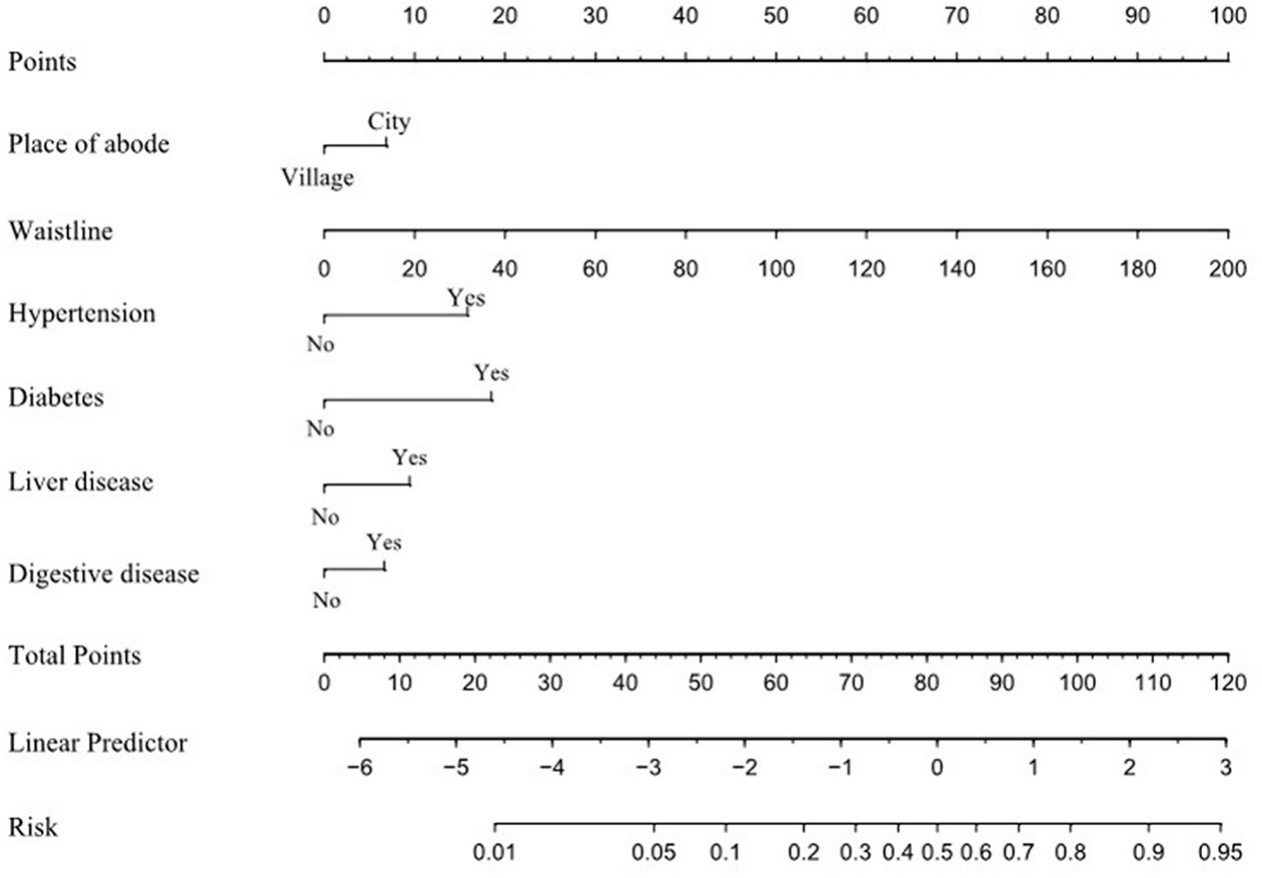
Nomogram.

A nomogram was created to illustrate the predictive model, enabling quantitative assessment of dyslipidemia (Figure 3). Risk was assessed by mapping each individual influencing factor onto the first row of the scale to derive a score for each factor, followed by summing the scores of the six factors to obtain a total score. Based on the total score, the predicted probability is obtained. A higher total score correlates with an increased risk of dyslipidemia.

### 3.3 Predictive model validation

#### 3.3.1 Discrimination

**Figure 4:**
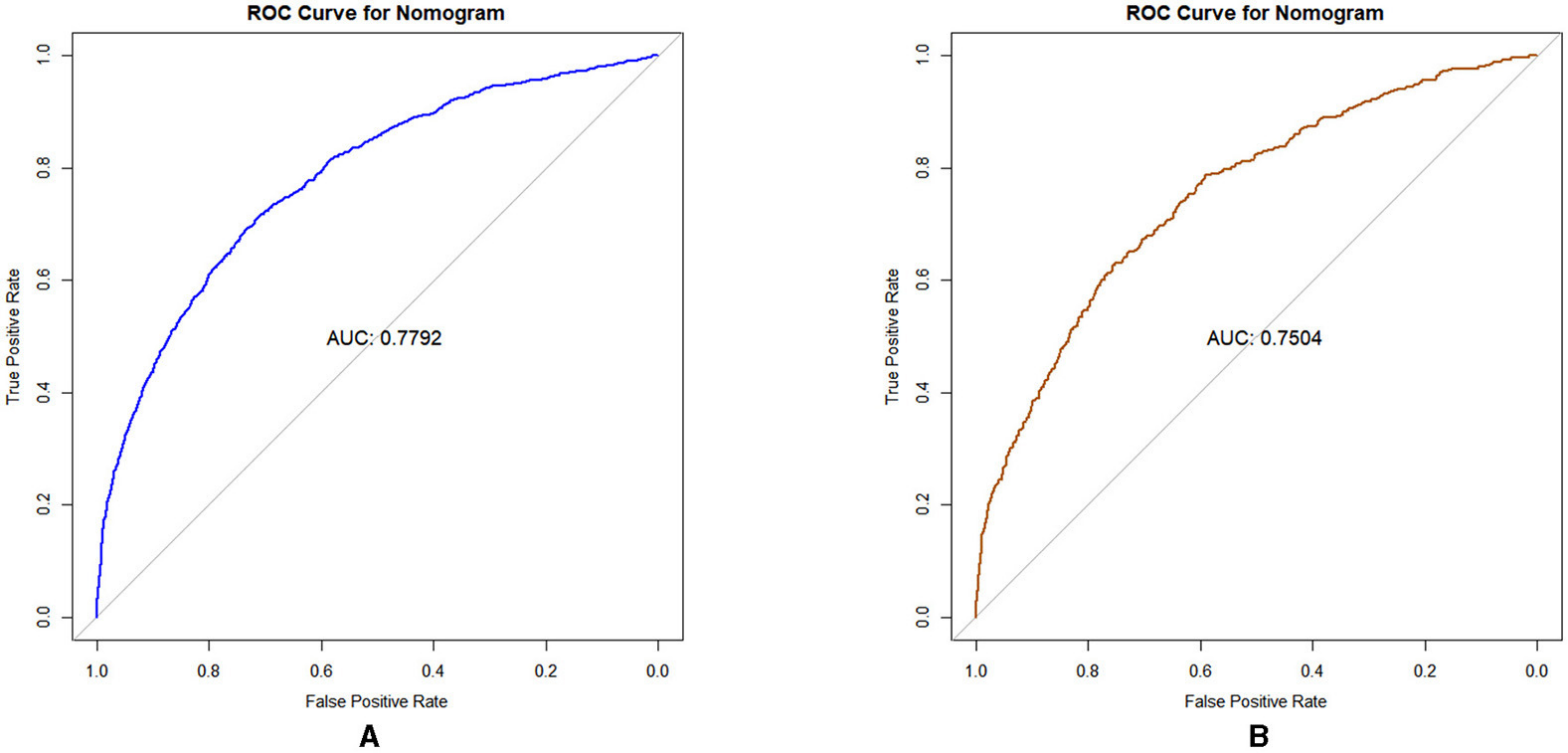
**(A)** The ROC for the training set; **(B)** The ROC for the validation set.

AUC used to assess the discrimination of prediction models for dyslipidemia risk among middle-aged and older adults living in the community. The results, shown in Figures 4A, B. Bindicate that the predictive model achieved an AUC value of 0.7792, with an accuracy of 88.7% in the training set. In the validation set, the AUC was also 0.7504, with an accuracy of 89.1%. These results indicate that the nomogram possesses a degree of discriminatory capability and predictive significance.

#### 3.3.2 Calibration of the predictive model

**Figure 5:**
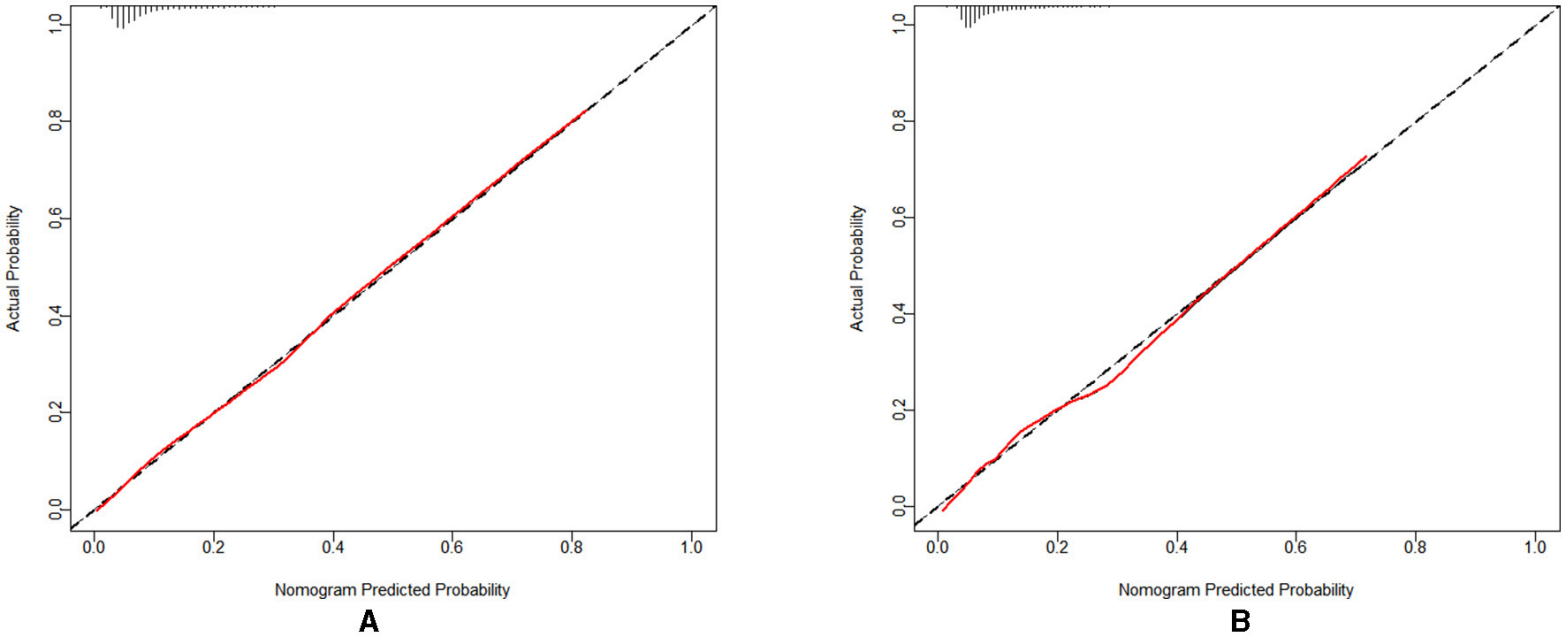
**(A)** The calibration curve for the training set; **(B)** The calibration curve for the validation set.

The nomogram's performance was assessed through a calibration plot, and the Hosmer–Lemeshow goodness-of-fit test (*P* > 0.05) demonstrates that the model shows a strong fit. The results of the tests indicated that the model exhibited a satisfactory fit for both the training set (χ^2^ = 9.532, df = 9, *P* = 0.391) and the validation set (χ^2^ = 5.847, df = 9, *P* = 0.7551). Figures 5A, B present the calibration plots for both the training and validation sets, derived from the Nomogram. The calibration curves for the nomogram illustrate a strong alignment between the predicted and observed probabilities of dyslipidemia in both the training set (Figure 5A) and the validation set (Figure 5B).

#### 3.3.3 Evaluation of clinical validity

**Figure 6:**
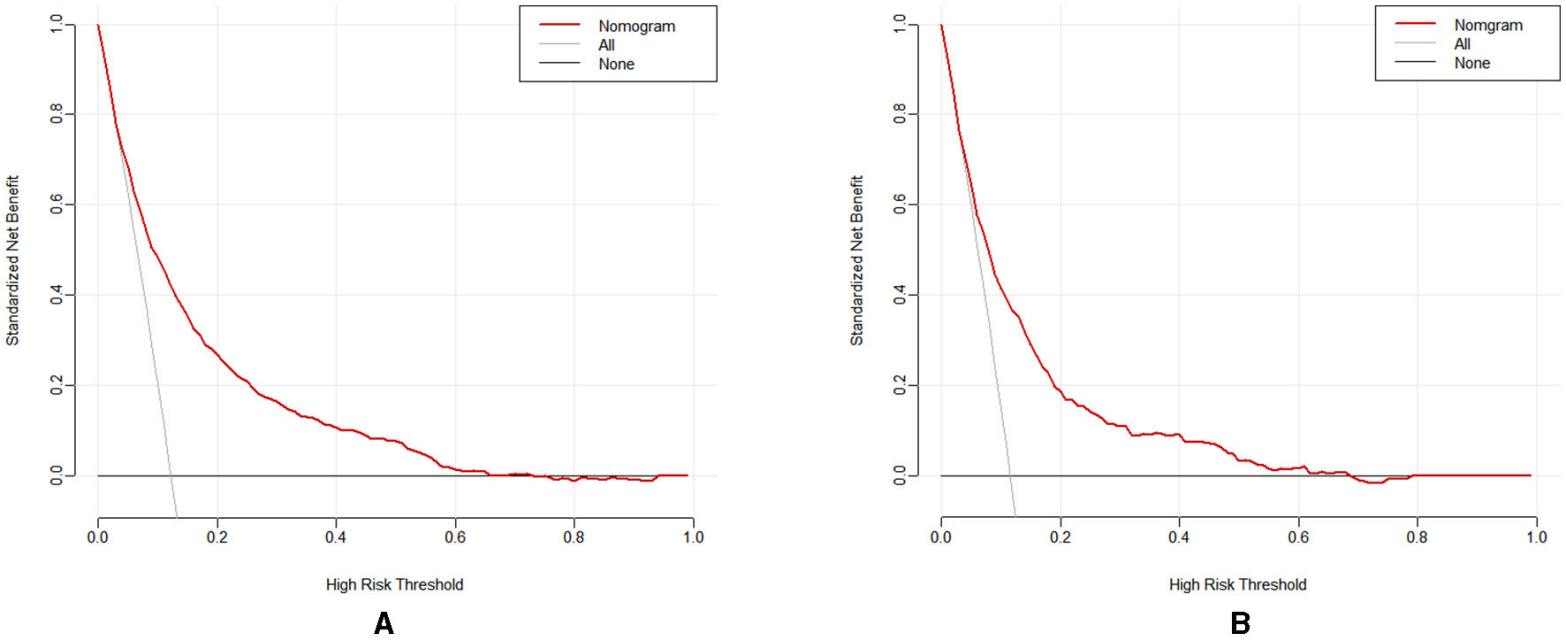
**(A)** The DCA for the training set; **(B)** The DCA for the validation set.

The clinical validity of the model was assessed through the DCA method, with the results presented in Figures 6A, B. The results of the study show that the net gains of the predictive model for the internal validation set mostly exceed the net gains for the two extreme cases, suggesting that the Nomogram model provides some net gains and predictive accuracy.

#### 3.3.4 Importance ranking of models

**Figure 7:**
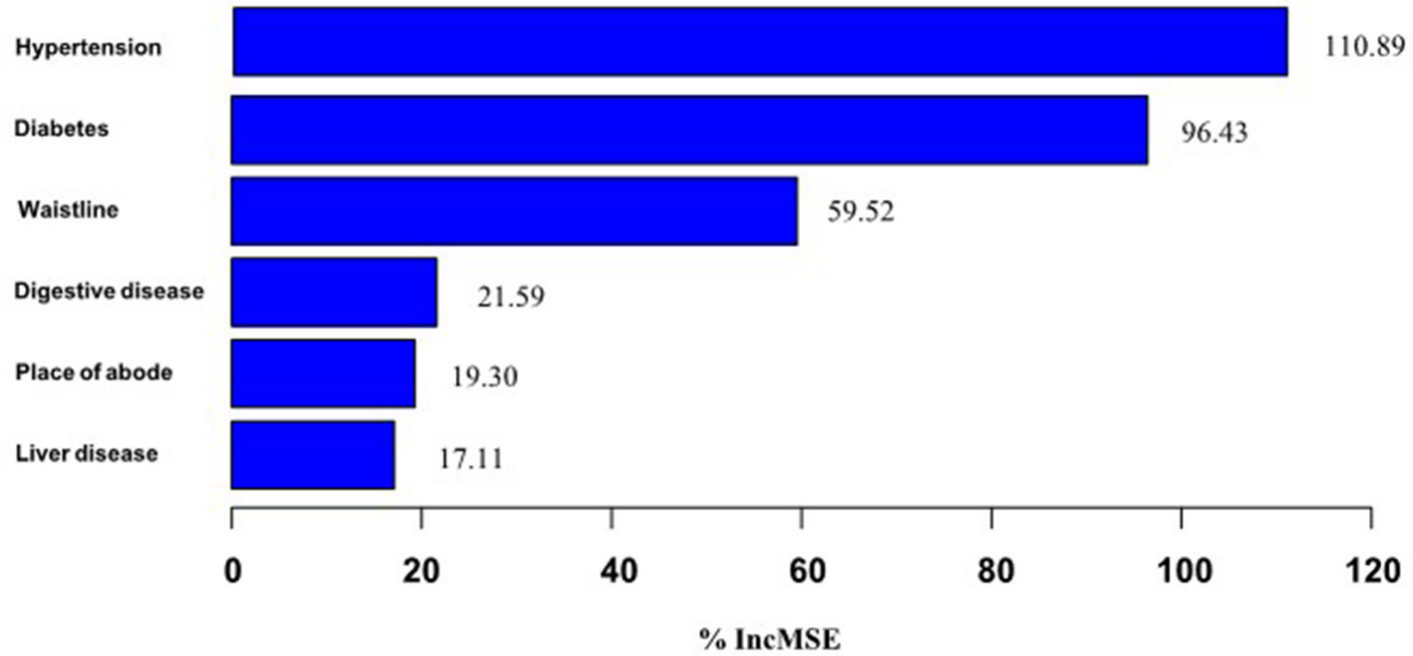
Ranking of factors in order of importance.

The importance of the risk factors is ranked using the random forest approach. And the results of the ranking are shown in Figure 7.

## 4 Discussion

The prevalence of dyslipidemia in Chinese middle-aged and older adults derived from the CHARLS was 12.03%. In this study, we constructed and validated a nomogram risk prediction model for dyslipidemia in Chinese middle-aged and older adults. The results of the model evaluation showed that the model had good differentiation, calibration, and clinical net benefit, which helped to accurately identify the risk of dyslipidemia in these people.

The model incorporated six influencing factors which included comorbid with hypertension, comorbid with diabetes or high blood sugar, waistline, comorbid with digestive disease, place of abode, and comorbid with liver disease. Among them, comorbid hypertension is the most important predictor of dyslipidemia in middle-aged and older adults. Hypertensive patients suffer from decreased vascular function due to elevated blood pressure that damages vascular endothelial cells, causing altered blood flow and microcirculation disorders, which in turn affects lipid metabolism (18). Insufficient insulin secretion in diabetic patients causes an increase in hormone-sensitive esterase activity, which leads to a large release of fatty acids and their accumulation in the body, causing dyslipidemia (19). Residents with excessive waistline are at increased risk of dyslipidemia due to excessive accumulation of abdominal fat, resulting in increased insulin resistance, decreased lipoprotein lipase activity, and increased TG and TC proteolipase activity (7). Patients suffering from digestive disease have weakened digestive function, leading to dysfunction in lipid digestion and absorption, which in turn affects blood lipid levels (20). A cross-sectional study of 37,263 consecutive healthy subjects undergoing routine health checkups showed that the correlation between H. *pylori* seropositivity and higher LDL-C and lower HDL-C levels was significant and independent (21). Urban residents have a high risk of dyslipidemia, which is different from the study of Niu Mixue et al. (22), probably because the level of economic development in urban areas is higher than in rural areas, the quality of life of the residents is better, the intake of high-fat food is more, and the amount of exercise is relatively small compared to the rural residents, and due to the faster pace of life, the social life of the pressures leading to greater mental stress (23). Patients with comorbid liver diseases are at increased risk of dyslipidemia due to liver function impairment, dull bile secretion impairment, decreased lipase secretion, imbalance between lipid synthesis and catabolism, and abnormal lipid transport. Liver disease is often accompanied by an inflammatory response, and inflammatory mediators such as TNF-alpha and IL-6 can affect lipid metabolism, leading to elevated lipid levels (24, 25). This suggests that middle-aged and older adults should take active monitoring and control of blood pressure and blood glucose, strengthen exercise, control weight, pay attention to dietary adjustments, and actively treat digestive and liver diseases to improve digestion and absorption functions to reduce the risk of dyslipidemia.

The innovation of this study is that first, we used nationally representative data from CHARLS 2015, which includes 12,589 Chinese middle-aged and older adults, which is more applicable to the Chinese middle-aged and older adults and makes the model more representative. Second, the model construction process strictly follows the PROBAST standard to control the risk of model bias as much as possible. Third, this study uses Lasso regression to compress the included variables further to make the model simpler and more practical. Fourth, this study used the Random Forest approach to rank the importance of the influencing factors and visualized them in a bar chart. Finally, the constructed model is visualized using Nomogram, which, through simple calculations, can provide a personalized assessment for each individual and is more conducive to screening needs. Its use as an efficient and accurate assessment tool can assist healthcare professionals in screening high-risk groups susceptible to dyslipidemia, providing a theoretical basis and entry point for developing early prevention and intervention measures. The prediction model has good clinical applicability and a broad prospect for use to help screen patients at high risk of dyslipidemia.

However, there are some limitations to this study: The model currently lacks external validation to verify the validity of the model and the constructed nomogram risk prediction model was developed based on data from middle-aged and older adults in China, whether the results of the study can be generalized to other regions and countries needs to be further validated using data from external cohorts. This study was retrospective, putting the modeling process at a high risk of bias. Variables in public databases may be limited by data collection and may not cover all factors that may be associated with dyslipidemia, such as dietary habits; and some of the factors are not explained and categorized in sufficient depth, which may affect the predictive ability of the model. The survey did not use longitudinal data, which could be considered in the future to optimize the nomogram model.

In the future, special studies can be carried out to analyze the relationship mechanism of dyslipidemia risk and to explore the interaction between different risk factors to construct more accurate risk prediction models. The model was externally validated through a multicenter study covering middle-aged and older adults in different geographic regions of China to verify the generalizability and applicability of the model. Developing intelligent risk assessment tools in collaboration with the engineering and technology fields to facilitate self-monitoring and risk assessment by middle-aged and older adults.

## 5 Conclusion

In this research, we constructed and validated a nomogram for assessing dyslipidemia in middle-aged and older adults. The model had a good performance. The developed nomogram is straightforward and easily comprehensible. The prediction model will be valuable in facilitating the screening and prediction of the risk of developing dyslipidemia in the middle-aged and older adults by community health organizations and clinical healthcare professionals.

## Data Availability

The raw data supporting the conclusions of this article will be made available by the authors, without undue reservation.
